# EP3 signaling is decoupled from the regulation of glucose-stimulated insulin secretion in β-cells compensating for obesity and insulin resistance

**DOI:** 10.1080/19382014.2023.2223327

**Published:** 2023-07-06

**Authors:** Michael D. Schaid, Jeffrey M. Harrington, Grant M. Kelly, Sophia M. Sdao, Matthew J. Merrins, Michelle E. Kimple

**Affiliations:** a Department of Medicine, Division of Endocrinology, Diabetes, and Metabolism, University of Wisconsin-Madison, Madison, WI, USA; b Research Service, William S. Middleton Memorial Veterans Hospital, Madison, WI, USA

**Keywords:** cAMP, Diabetes, EP3 receptor, Gɑ_z_, hyperglycemia, insulin secretion, prostaglandins, Rap1gap, β-cell

## Abstract

Of the β-cell signaling pathways altered by obesity and insulin resistance, some are adaptive while others contribute to β-cell failure. Two critical second messengers are Ca^2+^ and cAMP, which control the timing and amplitude of insulin secretion. Previous work has shown the importance of the cAMP-inhibitory Prostaglandin EP3 receptor (EP3) in mediating the β-cell dysfunction of type 2 diabetes (T2D). Here, we used three groups of C57BL/6J mice as a model of the progression from metabolic health to T2D: wildtype, normoglycemic *Leptin*^*Ob*^ (NGOB), and hyperglycemic *Leptin*^*Ob*^ (HGOB). Robust increases in β-cell cAMP and insulin secretion were observed in NGOB islets as compared to wildtype controls; an effect lost in HGOB islets, which exhibited reduced β-cell cAMP and insulin secretion despite increased glucose-dependent Ca^2+^ influx. An EP3 antagonist had no effect on β-cell cAMP or Ca^2+^ oscillations, demonstrating agonist-independent EP3 signaling. Finally, using sulprostone to hyperactivate EP3 signaling, we found EP3-dependent suppression of β-cell cAMP and Ca^2+^ duty cycle effectively reduces insulin secretion in HGOB islets, while having no impact insulin secretion on NGOB islets, despite similar and robust effects on cAMP levels and Ca^2+^ duty cycle. Finally, increased cAMP levels in NGOB islets are consistent with increased recruitment of the small G protein, Rap1GAP, to the plasma membrane, sequestering the EP3 effector, Gɑ_z_, from inhibition of adenylyl cyclase. Taken together, these results suggest that rewiring of EP3 receptor-dependent cAMP signaling contributes to the progressive changes in β cell function observed in the *Leptin*^*Ob*^ model of diabetes.

## GRAPHICAL ABSTRACT

**Figure:**
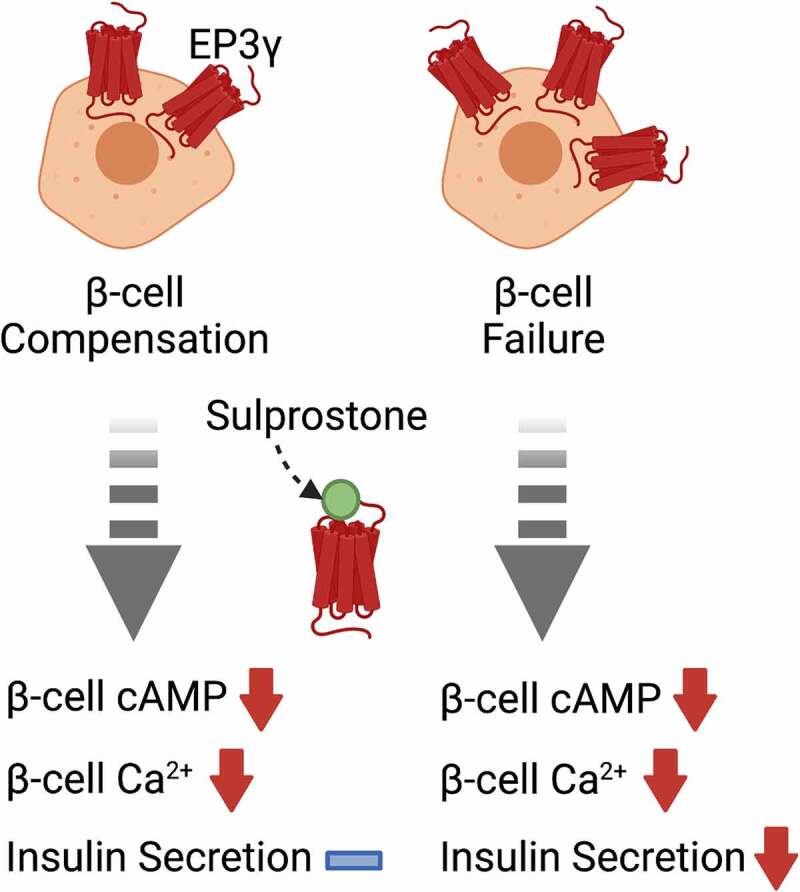

## Introduction

Type 2 diabetes (T2D) is associated with obesity and insulin resistance, yet not all insulin-resistant individuals are diabetic. Pancreatic β-cells can compensate via increased glucose sensitivity, mass, or both, resulting in circulating insulin levels sufficient to maintain normoglycemia. The second messenger cAMP plays a crucial role in β-cell compensation, and while many T2D therapies aim to increase β-cell cAMP levels, the efficacy of these therapies is not sufficient for many patients.^1^ Previous work by our group and others identified enhanced signaling through the prostaglandin EP3 receptor (EP3), a cAMP-inhibitory G protein-coupled receptor (GPCR) encoded by the *PTGER3* gene, in islets isolated from T2D mice and humans as compared to non-diabetic controls.^2–6^ The most abundant natural ligand of EP3 is prostaglandin E_2_ (PGE_2_), an eicosanoid derived from arachidonic acid whose islet production is similarly increased in the pathophysiological context of T2D.^3,4,7–10^ EP3 agonists reduce, while EP3 antagonists potentiate glucose-stimulated insulin secretion (GSIS) of islets from T2D mice and humans, while having little to no effect on islets from wildtype or non-diabetic subjects.^3,7,9^

In rat β-cell lines and primary pancreatic islets, EP3 is specifically coupled to G_z_, a member of the G_i/o_ subfamily of inhibitory G proteins that suppress cAMP production and GSIS.^3,11–16^ Yet, the role of EP3, its natural ligands, and effectors in T2D remains controversial. In the context of obesity, PGE_2_ and other arachidonic acid metabolites have tissue-specific beneficial effects on inflammation and insulin resistance, and EP3 knockout mice are more prone to metabolic dysfunction after consuming a high-fat diet.^17–19^ Furthermore, evidence exists for agonist-independent and cAMP-independent effects of EP3 receptor activity on cellular function and secretion processes.^20–22^ In this work, we examined the relationship of EP3 receptor expression and signaling on β-cell cAMP homeostasis, Ca^2+^ influx, and GSIS during the progression from metabolic health to β-cell compensation, and, finally, T2D. Using islets isolated from C57Bl/6J mice, either wildtype or homozygous for the *Leptin*^*Ob*^ mutation, in combination with high-sensitivity, temporal imaging of β-cell cAMP levels and Ca^2+^ oscillations, we find that increased EP3 receptor expression correlates directly with the effects of a selective EP3 agonist on cAMP production and Ca^2+^ influx, but that EP3 signaling in compensating β-cells is uncoupled from regulation of GSIS. Additionally, we confirm the coupling of the partially constitutively active EP3_γ_ variant is solely coupled to G_z_ to reduce β-cell cAMP and GSIS, while the effects of an EP3 antagonist on Ca^2+^ influx are G_z_-independent and have no effect on GSIS. Recruitment of an inactive form of Rap1GAP to the plasma membrane, where it sequesters the GTP-bound alpha subunit of G_z_, Gɑ_z_, provides a plausible explanation for the divergent effects of EP3 activity on β-cell second messenger pathways and GSIS in highly compensating NGOB islets.

## Results

### EP3 expression, but not PGE_2_ production, is associated with progression toward diabetes in islets from Leptin^Ob^ mice

**Figure 1.**
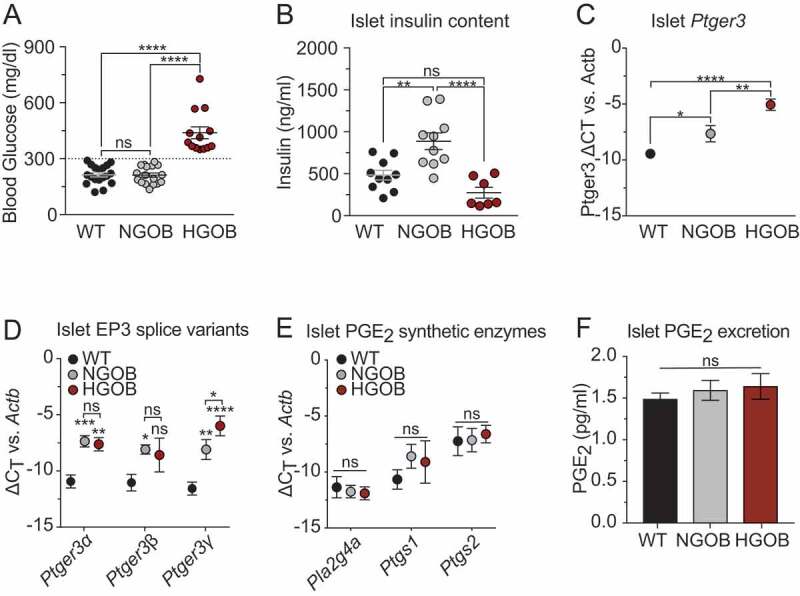
Expression of EP3 is associated with impaired insulin secretion independent of PGE_2_ production. (a) Blood glucose levels in C57Bl/6J wild-type (WT), normoglycemic Leptin^ob^ (NGOB) and hyperglycemic Leptin^ob^ (HGOB) mice. N = 13–18. (b) Islet insulin content. N = 7–10. (c) Relative Ptger3 transcript levels normalized to Actb. N = 9–15. (d) Relative Ptger3 isoform transcript levels normalized to Actb. N = 4–12. (e) Selected PGE_2_ synthetic enzymes (Pt2g4a, Ptgs1, and Ptgs2) as normalized to Actb. N = 4–12. (f) 24 h PGE_2_ excretion from cultured islets. N = 6–8. In all cases, N=individual mice or mouse islet preparations, and data represent the mean ± SEM. Data was analyzed by one-way ANOVA with Tukey test post-hoc (A-C, F) or multiple t-test corrected with Tukey test post-hoc (D,E). *P<.05; **P<.01; ***P<.001; and ****P<.0001. ns = not significant.

Using a random-fed blood glucose level of 300 mg/dl, two distinct populations of 10–12 week-old male C57Bl/6J *Leptin*^*Ob*^ mice were identified, both as compared to wild-type C57Bl/6J males – normoglycemic (NGOB) and hyperglycemic (HGOB) (Figure 1a). These results are consistent with a previous report plotting the relationship between plasma insulin and plasma glucose levels in a population of C57Bl/6J *Leptin*^*Ob*^ males.^23^ The mean random-fed blood glucose levels of wild type (WT) and NGOB were nearly identical (214 mg/dl and 212 mg/dl, respectively), whereas that of HGOB mice was just over twice as high (438.9 mg/dl, *p* < 0.0001). The mean islet insulin content was dramatically enhanced in NGOB as compared to WT islets, and trended lower in HGOB islets, consistent with T2D status (Figure 1b). EP3 (*Ptger3*) mRNA abundance was significantly higher in islets from both NGOB and HGOB mice as compared to WT (Figure 1c).

The mouse *Ptger3* gene encodes three EP3 splice variants, EP3α, EP3β, and EP3γ, that differ only in their cytoplasmic C-terminal tail, a region important for properties including G protein coupling and constitutive activity.^11,24^ Using isoform-specific qPCR primers, the mean EP3α and EP3β mRNA abundance was modestly but significantly elevated in both NGOB and HGOB islets as compared to WT controls (Figure 1d, *left* and *middle*). However, EP3γ mRNA abundance exhibited the same stepwise increase from WT to NGOB to HGOB as observed with total *Ptger3* expression (Figure 1d, *right*). Neither NGOB nor HGOB mouse islets had any significant alteration in the mRNA abundance of the PGE_2_ synthetic enzymes important in the β-cell^3,7^ as compared to WT controls (Figure 1e). Consistent with a lack of upregulation of important PGE_2_ synthetic genes, islet PGE_2_ excretion was nearly identical among the three groups (Figure 1f).

### Validation of the cAMP FRET assay and Leptin^Ob^ islet phenotypes

**Figure 2.**
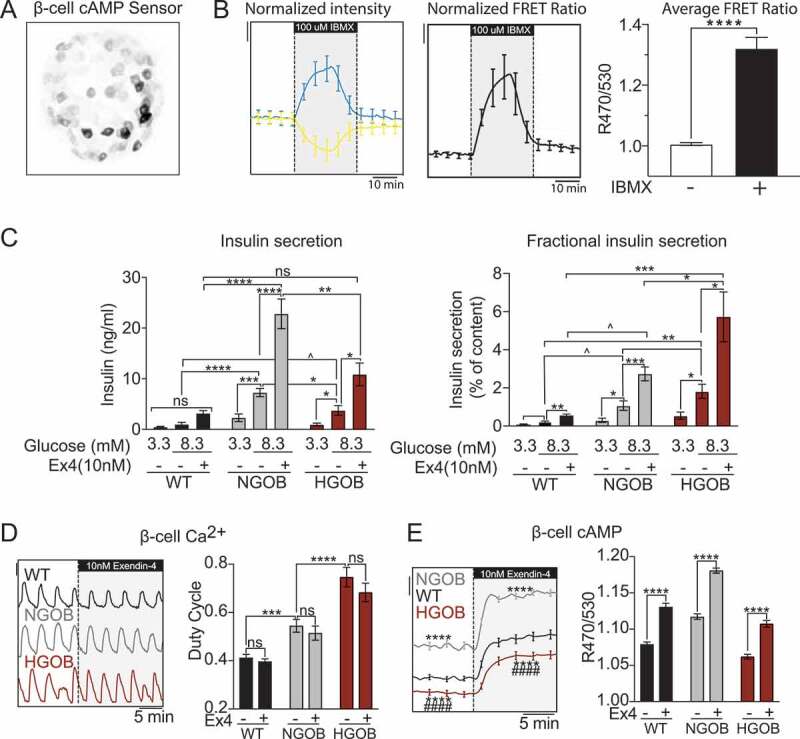
Validation of β-cell-specific cAMP FRET sensor and correlation of β-cell cAMP levels with islet pathophysiology. (a) 3D projection of WT islets expressing the β-cell specific cAMP biosensor (Epac-S^H187^) as recorded by two-photon microscopy. (b) Normalized fluorophore intensity (left), FRET ratio (middle) and average FRET ratio (right) from WT islets expressing cAMP biosensor treated with 100 μM 3-Isobutyl −1-methylxanthine (IBMX). N = 7 islets. Scale bar = 0.02. (c) Glucose stimulated insulin secretion from WT, NGOB and HGOB islets treated with 3.3 mM glucose or 8.3 mM glucose ±10 nM E x 4. Data is represented as both total insulin secreted (left) and fractional insulin secretion as a percent of total insulin content. N = 7–10 mice. (d) Representative Ca^2+^ recordings and duty cycle quantification of WT, NGOB and HGOB islets treated with 9 mM glucose followed by 9 mM glucose +10 nM E x 4. Scale bar = 0.01. (e) Representative average cAMP recordings and quantification of WT, NGOB, and HGOB islets treated with 9 mM glucose followed by 9 mM glucose +10 nM E x 4. N = 55–72 islets from three mice (NGOB and HGOB) and 90 islets from six mice (WT). Scale bar = 0.025. Error bars represent the standard error of the mean (SEM). Data was analyzed by one-way ANOVA with Tukey test post-hoc within groups and multiple T-test with Tukey test post-hoc among groups.*p < 0.05, **p < 0.01, ***p < 0.001, ****p < 0.0001. For panel E, ****p < 0.0001 as compared to WT and ^####^*p* <0.0001 as compared to NGOB.

We utilized a high-affinity cAMP FRET biosensor (Epac-S^H187^)^25^ adenovirally-expressed under the control of the insulin promoter in order to confer β-cell specificity.^26^ A representative two-photon microscopy image of a transduced islet shows penetrance of the expression into the interior of the islet (Figure 2a). Transduced islets perfused with imaging buffer showed a dramatic increase in the FRET ratio in response to the phosphodiesterase inhibitor 3-isobutyl−1-methylxanthine (IBMX, 100 µM) (Figure 2b, *left*), reflecting an increase in the level of cytosolic cAMP (Figure 2b, *middle and right)*.

Exendin−4 (Ex4) is a stable GLP−1 receptor agonist that potentiates GSIS primarily by increasing cAMP.^1^ Therefore, E×4was used to both quantify the secretion phenotype of islets from WT, NGOB, and HGOB mice and confirm its relationship with β-cell cAMP levels. Consistent with their β-cell compensation phenotype, islets from NGOB mice secrete significantly more insulin in 8.3 mM glucose, the threshold at which cAMP potentiates GSIS,^27^ than 3.3 mM glucose. Furthermore, their mean GSIS response is significantly higher than WT, whether represented as total insulin secreted or insulin secreted as a percent of content (Figure 2c). Islets from HGOB mice secrete significantly less insulin in 8.3 mM glucose than NGOB, and their fractional GSIS response was higher (Figure 2c), consistent with their T2D phenotype. All three groups responded strongly to exendin−4 (Ex4) to potentiate GSIS by approximately 2-fold over 8.3 mM glucose alone, whether represented as total or fraction of content (Figure 2c). Imaging experiments performed in parallel with GSIS assays reveal that Ca^2+^ duty cycle (fractional time in the active phase of an oscillation)^28^ was sequentially increased in NGOB and HGOB islets as compared to WT, but was unaffected by E×4in any group (Figure 2d). In contrast, β-cell cAMP was strongly and significantly increased in NGOB islets as compared to WT and reduced in HGOB islets as compared to the other groups (Figure 2e). In all three cases, consistent with GSIS results, E×4significantly increased cAMP levels (Figure 2e).

### Endogenous agonist-dependent EP3 signaling does not contribute to β-cell compensation or dysfunction in C57Bl/6J Leptin^Ob^ islets

**Figure 3.**
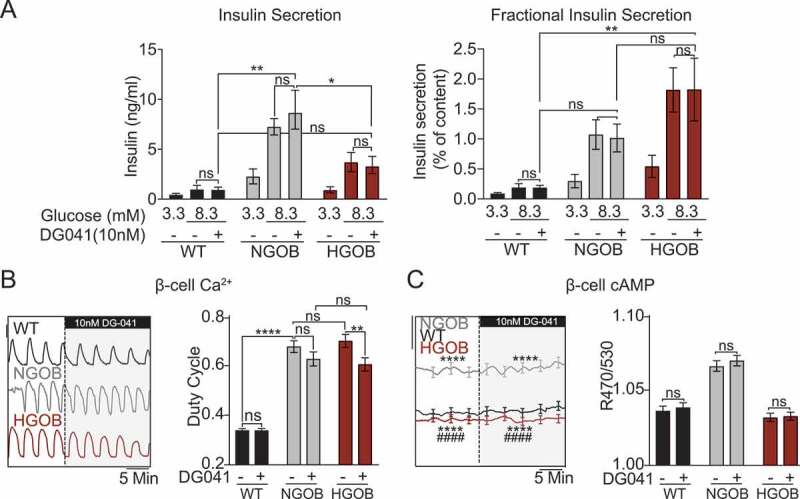
Agonist-sensitive EP3 signaling does not influence WT, NGOB, or HGOB β-cell cAMP production or function. (a) Glucose stimulated insulin secretion from WT, NGOB and HGOB islets treated with 3.3 mM glucose or 8.3 mM glucose ±10 nM DG041. Data are represented as both total insulin secreted (left) and fractional insulin secretion as a percent of total insulin content. N = 7–10 mice. (b) Representative Ca^2+^ recordings and duty cycle quantification of WT, NGOB and HGOB islets treated with 9 mM glucose followed by 9 mM glucose +10 nM DG041. Scale bar = 0.01. (c) Representative average cAMP recordings and quantification of WT, NGOB and HGOB islets treated with 9 mM glucose followed by 9 mM glucose +10 nM DG041. Scale bar = 0.025. In (B) and (C), N = 55–72 islets from three mice (NGOB and HGOB) and 108 islets from six mice (WT). Error bars represent the standard error of the mean (SEM). Data was analyzed by one-way ANOVA among groups or multiple T-test by treatment, both with Tukey test post-hoc.*p < 0.05, **p < 0.01, ***p < 0.001, ****p < 0.0001. For panel C, ****p < 0.0001 as compared to WT and ^####^*p* <0.0001 as compared to NGOB.

Both PGE_2_ production and EP3 expression have previously been shown to contribute to βcell dysfunction in the BTBR *Leptin*^*Ob*^ mouse model, a unique and robust model of T2D, as well as human islets isolated from T2D human organ donors.^3^ In the current work, islet PGE_2_ synthetic gene expression and PGE_2_ excretion are both unchanged in NGOB or HGOB islets vs. WT on the C57Bl/6J background (Figure 1e,f), suggesting agonist-sensitive EP3 signaling does not contribute to either β-cell compensation or β-cell failure in this model. To confirm this at the molecular level, we utilized the EP3-specific antagonist, DG041 (10 nM), which blocks binding of endogenous PGE_2_ to EP3. As expected, DG041 had no effect on GSIS (Figure 3a), Ca^2+^ duty cycle (Figure 3b), or cAMP levels (Figure 3c).

*EP3-dependent suppression of β-cell cAMP is decoupled from changes in insulin secretion in compensating but not decompensating Leptin*^*Ob*^ *islets*

**Figure 4.**
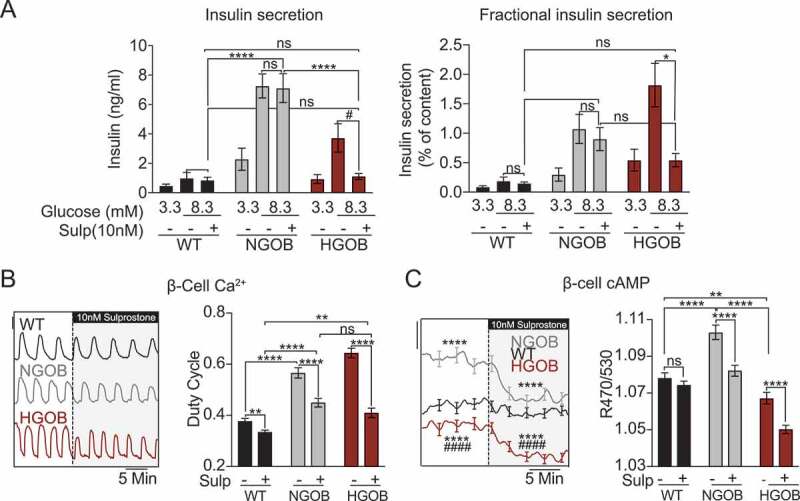
The EP3 agonist sulprostone reduces Ca^2+^ duty cycle and cAMP levels but inhibits insulin secretion only in failing β-cells. (a) Glucose stimulated insulin secretion from WT, NGOB and HGOB islets treated with 3.3 mM glucose or 8.3 mM glucose ±10 nM sulprostone. Data is represented as both total insulin secreted (left) and fractional insulin secretion as a percent of total insulin content. *N* = 7–10 mice. (b) Representative Ca^2+^ recordings and duty cycle quantification of WT, NGOB and HGOB islets treated with 9 mM glucose followed by 9 mM glucose +10 nM sulprostone. Scale bar = 0.01. (c) Representative average cAMP recordings and quantification of WT, NGOB and HGOB islets treated with 9 mM glucose followed by 9 mM glucose +10 nM sulprostone. Scale bar = 0.025. In (B) and (C), *N* = 55–82 islets from three mice (NGOB and HGOB) and 84 islets from six mice (WT). Error bars represent the standard error of the mean (SEM). Data was analyzed by one-way ANOVA among groups or multiple T-test by treatment, both with Tukey test post-hoc.*p < 0.05, **p < 0.01, ***p < 0.001, ****p < 0.0001. For panel C, ****p < 0.0001 as compared to WT and ^####^ *p* < 0.0001 as compared to NGOB.

The EP3γ splice variant is approximately 80% constitutively active, meaning it does not require PGE_2_ binding to signal downstream.^29,30^ Yet, as it remains partially agonist-sensitive, the EP3-selective agonist, sulprostone, can be utilized to explore its downstream effects. Consistent with previous reports using islets from lean and/or non-diabetic mice and humans,^3,7,11,17^ sulprostone had no effect on GSIS in WT or NGOB islets, but reduced GSIS in HGOB islets (Figure 4a). Interestingly, however, sulprostone reduced the Ca^2+^ duty cycle in all three groups, most strikingly in NGOB and HGOB (Figure 4b), and significantly reduced cAMP levels in NGOB and HGOB β-cells (Figure 4c).

### Gaz loss potentiates β-cell cAMP levels and GLP1R responsiveness

**Figure 5.**
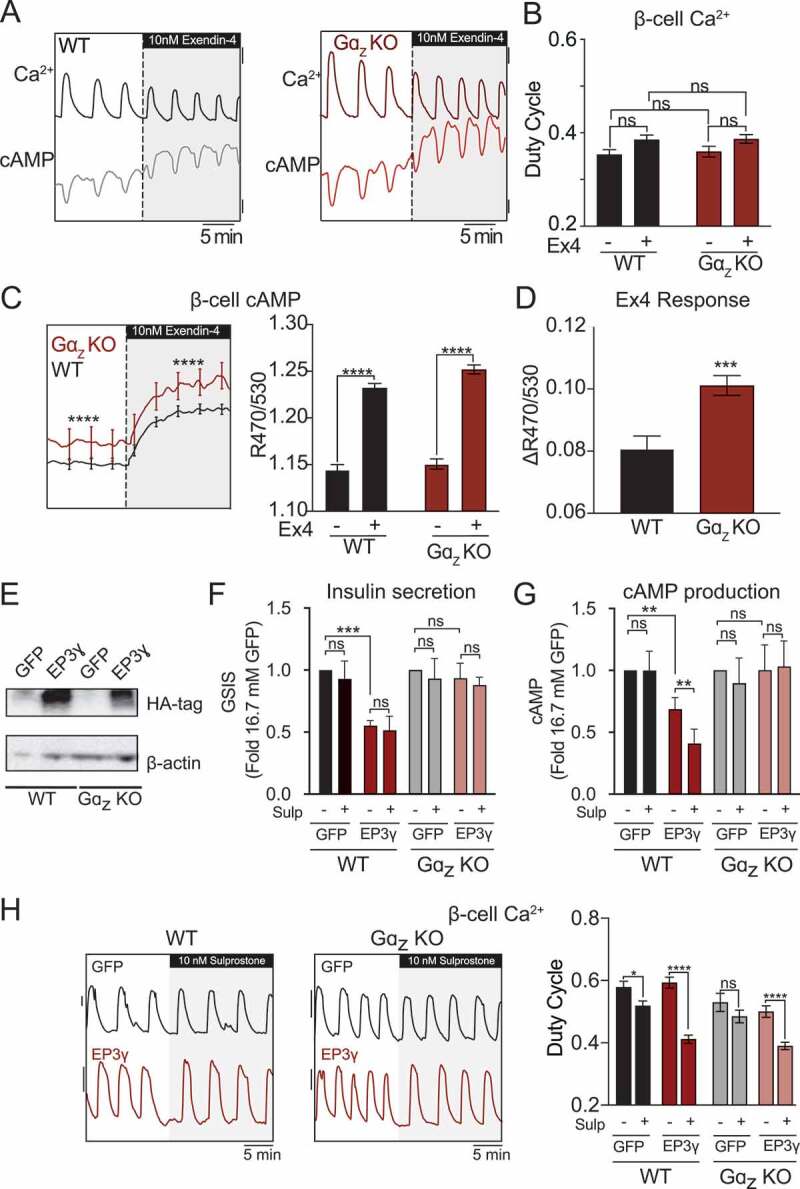
Ga_z_-null islets have higher cAMP levels and E x 4 response, and EP3γ reduces cAMP production and GSIS or Ca^2+^ duty cycle in a Gɑ_z_-dependent or -independent manner, respectively. (a) Representative simultaneous recordings of Ca^2+^ and cAMP in WT and Gα_z_ KO islets treated with 9 mM glucose followed by 9 mM glucose +10 nM exendin − 4 (E x 4). Scale bars: Ca^2+^ = 0.1 and R470/530 (cAMP) = 0.02. (b) Quantification of islet Ca^2+^ duty cycle for the data shown in (A). (c) Average temporal (left) and pre/post E x 4 (right) cAMP levels for the experiments represented in (A). (d) Islet E x 4 response, as calculated by normalizing the mean cAMP levels in 9 mM glucose + E x 4 to those in 9 mM glucose alone. In A-D, *N* = 67–72 islets from three mice each. (e) Representative Western blot of WT and Gɑ_z_ knockout (KO) C57Bl/6N islets transduced with an adenovirus encoding HA-tagged EP3γ or a GFP control adenovirus. (f) Insulin secreted in 16.7 mM glucose from cultured islets isolated from WT or Gα_z_ KO mice expressing EP3_γ_ or GFP control, with and without 10 nM sulprostone. Within each experiment, the GSIS response of a group was normalized to that of its GFP control. *N* = 3 independent experiments. Figure adapted from Schaid et al., 2021.^31^ (g) cAMP production from cultured islets isolated from WT or Gα_z_ KO mice expressing EP3_γ_ or GFP control, with and without 10 nM sulprostone. Within each experiment, the GSIS response of a group was normalized to that of its GFP control. Figure adapted from Schaid et al., 2021.^31^ (h) Representative recordings of Ca^2+^ oscillations (left) and duty cycle quantification (right) in islets isolated from WT or Gα_z_ KO mice expressing EP3_γ_ or GFP control, with and without 10 nM sulprostone. *N* = 3 independent experiments. In all panels, error bars represent the standard error of the mean (SEM). Data was analyzed by two-way ANOVA with Tukey test post hoc (A-E) or multiple unpaired t-test corrected with Holm-Sidak test post-hoc, or unpaired t-test (F). *p < 0.05, **p < 0.01, ***p < 0.001, and ****p < 0.0001.

Islets from Gɑ_z_-null mice have significantly higher cAMP production and enhanced potentiation of GSIS in response to Ex4.^12,27,31^ In order to more precisely study the molecular mechanisms downstream of Gɑ_z_, islets from wild-type and Gα_z_-null C57Bl/6N mice were stimulated with 9 mM glucose followed by the addition of 10 nM Ex4, and Ca^2+^ and cAMP oscillations recorded. Representative cAMP and Ca^2+^ traces from one experiment are shown in Figure 5a. While the Ca^2+^ duty cycle was unaffected by genotype or E×4treatment (Figure 5b), Gα_z_-null β-cells displayed significantly higher basal and Ex4-stimulated cAMP levels (Figure 5c). Normalizing the cAMP responses of Ex4-treated islets to that of glucose alone confirms the enhanced GLP1R responsiveness of Gα_z_-null β-cells (Figure 5d).^31^

### EP3γ suppresses β-cell cAMP production and GSIS exclusively through Gα_z_, while its agonist-dependent effects on Ca^2+^ duty cycle are Ga_z_-independent

Our previous work with HGOB mouse islets revealed that sulprostone inhibits GSIS in a pertussis toxin-insensitive manner, confirming the EP3 receptor is exclusively coupled to Gα_z_.^12^ The EP3_γ_ splice variant, which has significant constitutive activity,^30,32–34^ is the most highly differentially expressed EP3 splice variant in WT, NGOB, and HGOB islets. To determine the effects of EP3_γ_ on β-cell second messenger signaling and downstream GSIS, WT and Gɑ_z_-null islets were transduced with an adenovirus encoding HA-tagged EP3_γ_ that bicistronically expresses GFP, or a GFP control adenovirus, followed by GSIS, cAMP production, and Ca^2+^ imaging assays. Western blot analysis for the HA-tag confirmed EP3_γ_ overexpression (Figure 5e). In stimulatory glucose, EP3_γ_ expression alone reduced β-cell GSIS, with sulprostone having no additional effect (Figure 5f). Because the EP3_γ_ adenovirus expresses a GFP tracer, cAMP imaging assays could not be performed. Therefore, cAMP production was measured by ELISA, with the addition of IBMX to block cAMP degradation. In WT islets, EP3_γ_ overexpression alone also reduced cAMP production, with sulprostone having an additional effect (Figure 5g). This reduction in cAMP production was ablated in Gɑ_z_-null islets (Figure 5g). Finally, Ca^2+^ imaging was performed to determine the effects of constitutive and agonist-dependent EP3_γ_ activity on Ca^2+^ duty cycle (Representative traces are shown in Figure 5h, *left*). EP3_γ_ expression alone had little impact on Ca^2+^ duty cycle in either WT or Gɑ_z_-null islets expressing GFP, but when EP3_γ_was overexpressed in WT islets, we observed a dramatic sulprostone-mediated reduction in Ca^2+^ duty cycle (Figure 5h, *right*). These effects were completely Gɑ_z_-independent, as nearly identical results were observed in EP3_γ_-transduced Gɑ_z_-null islets (Figure 5h, *right*).

### Membrane-associated Rap1GAP and Phospho-PKA substrate levels are correlated with elevated cAMP levels in NGOB islets

**Figure 6.**
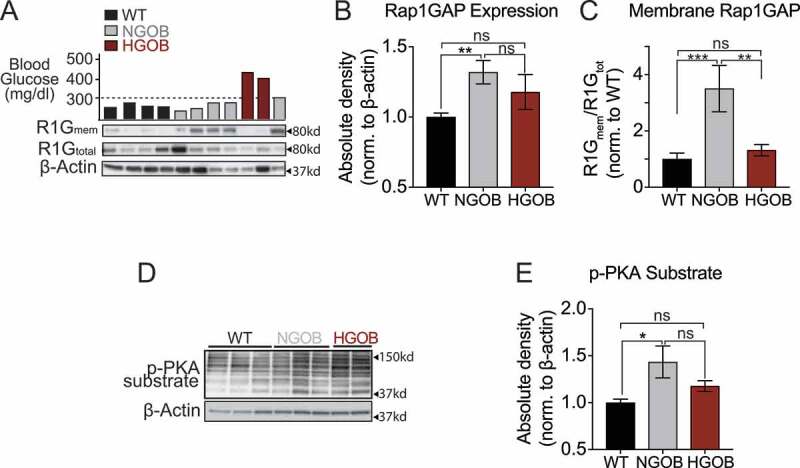
Expression and membrane localization of the Gα_z_ effector, Rap1GAP, is associated with higher cAMP signaling in islets from highly compensating NGOB mice. (a) Representative Western blot of Rap1GAP (total cellular and membrane-associated) and β-actin in WT, NGOB and HGOB islets. (b) Quantification of total cellular Rap1GAP in WT, NGOB and HGOB islets, as normalized to β-actin. (c) Quantification of membrane-associated Rap1GAP in WT, NGOB and HGOB islets, as normalized to β-actin. (d) Representative Western blot of phospho-PKA substrate in WT, NGOB, and HGOB islets. (e) Quantification of phospho-PKA substrate as normalized to β-actin. In (B), (C), and (E), data were normalized to relative WT expression. *n* = 4–10 mice/group. Error bars represent the standard error of the mean (SEM). In (B), (C), and (E), data was analyzed with one-way ANOVA with Tukey test post hoc *p < 0.05, **p < 0.01, ***p < 0.001.

Besides adenylyl cyclase, a primary downstream effector of Gα_z_ is Rap1GAP, a negative regulator of the small G protein, Rap1.^35,36^ Protein kinase A (PKA) and the Rap1 activator, Exchange protein activated by cAMP (Epac), are the two primary cAMP effectors and are important players in β-cell proliferation and the amplifying pathway of insulin secretion.^37–39^ Importantly, the binding of Gα_z_ to adenylate cyclase and Rap1GAP are mutually exclusive, and Rap1GAP expression partially relieves the inhibitory constraint that constitutively active Gα_z_ places on cAMP production.^35,36^ As a first step in determining the relevance of Gɑ_z_/Rap1GAP binding to the phenotype of WT, NGOB, and HGOB islets, Rap1GAP Western blot analysis was performed on total cellular and islet membrane lysates. A representative Western blot image is shown in Figure 6a. Rap1GAP protein abundance was moderately elevated in NGOB islets as compared to WT (Figure 6b), but the proportion of membrane localized Rap1GAP as a fraction of total cellular Rap1GAP was dramatically enhanced, an effect that was ablated in HGOB islets (Figure 6c). Additionally, Rap1GAP is PKA-phosphorylated at Ser−441 and Ser−0449, ablating its GAP activity.^40,41^ As cAMP levels were dramatically elevated in NGOB islets as compared to WT (Figure 3c), we hypothesized PKA activity would similarly be enhanced – an effect confirmed by Phospho-PKA substrate assay (Figure 6d,e).

## Discussion

β-cell cAMP is essential for normal cellular function, and impaired β-cell cAMP levels in diabetes pathology have been attributed to alterations in receptor regulated Gα_s_ activity.^26,42–47^ In this study, we demonstrate a robust increase in cytoplasmic β-cell cAMP and insulin secretion in islets from NGOB mice as compared to wild-type, lean controls that is lost in islets from HGOB mice. While β-cell cAMP levels are generally more predictive of glucose-stimulated insulin secretion than Ca^2+^ influx, which is permissive, the EP3-dependent suppression of β-cell cAMP was decoupled from changes in insulin secretion from compensating NGOB islets. In contrast, both cAMP and GSIS were effectively reduced by EP3 agonism in HGOB islets. These changes in β-cell function were mirrored by changes in EP3 expression, providing further evidence that EP3 signaling is linked to diabetes progression in the *Lep*^*Ob/Ob*^ mice. We also provide evidence for Gɑ_z_-dependent and -independent effects of EP3_γ_ signaling, which, again, have differential effects on β-cell second messenger pathways and GSIS. Finally, we propose activated Gɑ_z_ in NGOB islets binds and recruits inactive Rap1GAP to the plasma membrane, where it effectively sequesters Gɑ_z_ from both EP3 and adenylyl cyclase, explaining the enhanced basal cAMP levels and GSIS in NGOB islets as compared to WT and HGOB islets.

PGE_2_ is the most abundant natural ligand of EP3 and is produced from arachidonic acid cleaved from plasma membrane phospholipids by phospholipase A_2_ (PLA_2_) isozymes. The synthesis of arachidonic acid to PGE_2_ is rate-limited by the activity of prostaglandin endoperoxidase synthase (PTGS) 1 and 2 (a.k.a. COX 1 and 2), which catalyze the intermediate conversion of PGH_2_ to PGE_2_. Elevated islet expression of specific PGE_2_ synthetic enzymes and/or islet PGE_2_ excretion have been observed in islets from T2D animals and humans.^2,4,9,10,48^ Yet, in this study, PGE_2_ production was not enhanced in HGOB islets above the levels observed in either lean or NGOB. Supporting this, the specific EP3 antagonist, DG041, had no effect on cAMP production or GSIS in HGOB islets. These findings are in contrast to our own previously-published results with islets from *Lep*^*Ob/Ob*^ mice in the BTBR strain background – a very robust model of T2D caused by β-cell dysfunction and loss of functional β-cell mass that occurs with 100% penetrance by 10 weeks of age.^3,7^ In the C57Bl/6J *Leptin*^*Ob*^ line, it is possible chronic hyperglycemia, over time, would lead to enhanced PGE_2_ synthetic gene expression and up-regulated agonist-dependent EP3 signaling. Yet, the results presented here suggest up-regulation of EP3 itself is the largest contributor to β-cell failure in HGOB islets.

The mouse *Ptger3* gene encodes for three splice variants, EP3α, EP3β and EP3γ, all with varying degrees of constitutive activity. EP3γ has approximately 80% constitutive activity for coupling to G_i/o_ subfamily proteins,^29,32^ and it is this splice variant whose expression is sequentially altered in WT, NGOB, and HGOB islets. Not surprisingly, we find EP3γ expression correlates with impaired cAMP levels in HGOB islets. The fact sulprostone significantly reduces NGOB β-cell cAMP and Ca^2+^ levels without significantly affecting GSIS was unexpected, and to our knowledge, is the first report of decoupling second messenger signaling with insulin secretion. Using islets from WT and Gɑ_z_-null mice, we found that constitutively active EP3_γ_ is solely coupled to Gɑ_z_ to reduce cAMP production and GSIS, with no additional effect of sulprostone on GSIS. In contrast, ligand-activated EP3 reduced both cAMP production and Ca^2+^ duty cycle, the latter in a Gɑ_z_-independent manner; again, with no effect on GSIS. These findings at least partially confirm the molecular mechanisms underlying EP3_γ_ signaling and show the divergence of the effects of EP3_γ_ on cAMP production, Ca^2+^ duty cycle, and GSIS are reproducible.

At first, the up-regulation of EP3_γ_ expression in NGOB islets was perplexing, as NGOB islets have enhanced cAMP production and GSIS as compared to WT islets, not reduced. Based on the traditional role of G_i/o_ proteins to block adenylyl cyclase activity and reduce cAMP production, EP3γ activity may reduce NGOB β-cell cAMP levels from an even higher theoretical baseline. Yet, numerous signaling changes have been demonstrated in β-cell compensation and failure, such as those mediated by β-cell stress pathways,^4,6,10,49^ many of which converge on cAMP.^14,42,50–52^ In this scenario, EP3γ up-regulation may be a compensatory adaptation to dampen β-cell stress and promote β-cell survival. In contrast, molecular signaling mechanisms and their ultimate biological effects may differ in the healthy, compensating, and failing β-cell based on the (patho)physiological milieu in which the islet exists. Therefore, it is possible that the primary effector of constitutively active EP3γ is not adenylyl cyclase, and that shuttling of EP3γ to this alternate effector might even contribute to the elevated cAMP level of NGOB β-cells. Without an allosteric EP3 antagonist or a β-cell-specific EP3γ C57Bl/6J *Leptin*^*Ob*^ knockout mouse, these models cannot be differentiated. We, though, favor a third model. In addition to adenylyl cyclase, GTP-bound Gɑ_z_ can bind to Rap1GAP in a mutually exclusive manner. The half-time for Gɑ_z_ GTP hydrolysis is ~10 min, meaning, once it is activated, it acts as a partial tonic inhibitor of cAMP production.^13^ We propose Rap1GAP recruitment effectively sequesters Gɑ_z_ from adenylyl cyclase, potentiating cAMP production and explaining the elevated baseline cAMP levels in NGOB islets. Increased cAMP production would also enhance the activity of PKA and Epac, both which are known to stimulate GSIS and β-cell replication.^37^ While we did not study Rap1 signaling in this work, we found increased Phospho-PKA substrate abundance in islets from NGOB mice. As Rap1GAP can be phosphorylated and inactivated by PKA,^40^ membrane-associated Rap1GAP would be unable to reduce the potentiating effects of Rap1 on β-cell proliferation and GSIS. Since Rap1 is essentially constitutively active in the absence of a GAP protein,^53^ we propose increased EP3_γ_ expression in NGOB islets likely relieves Gɑ_z_’s tonic inhibitory constraints on critical β-cell signaling pathways, explaining both the increased GSIS and β-cell mass in islets highly compensating for obesity and insulin resistance. Confirming this model is the subject of much current research.

In both NGOB islets and islets from WT and Gɑ_z_-null mice transduced with EP3_γ_ adenovirus, sulprostone strongly reduced Ca^2+^ duty cycle, with no effects on GSIS. While the precise mechanisms underlying these effects have not been determined, one possible explanation is that ligand-bound EP3_γ_ is internalized, where it regulates calcium-induced calcium release. Soluble adenylyl cyclase (sAC, a.k.a. Adcy10) is localized at intracellular membranes and requires both Ca^2+^ and bicarbonate binding for its activity. Interestingly, sAC has also been identified as a critical mediator of β-cell compensation.^54^ As increased mitochondrial bicarbonate production would only be observed under conditions of metabolic overload, sAC activity would only be observed in NGOB and HGOB islets. Yet, if this model holds true, it does not explain the difference in sulprostone’s effects on cAMP, Ca^2+^ duty cycle, and GSIS in NGOB and HGOB islets. We speculate, but cannot confirm, cAMP produced by sAC regulates β-cell proliferation or survival in compensating β-cells but is harnessed to regulate insulin secretion in the T2D state. Future research to confirm this model, and the role of EP3_γ_ in regulating sAC activity, is currently ongoing.

In summary, previous work has implicated the β-cell EP3 receptor as a potential target for T2D therapeutics, but our results with the C57Bl/6J *Leptin*^*Ob*^ mouse model suggest that the contribution of EP3 to β-cell compensation and function is much more nuanced. One limitation of this study is that whole islets were used; therefore, we cannot exclude that EP3 has a role in other islet cell types that influence β-cell second messenger signaling and GSIS. In a β-cell line, though, EP3 expression correlates directly with the effects of PGE_2_ and sulprostone on GSIS, and EP3 is the sole prostanoid family receptor that negatively regulates insulin secretion.^55^ A second limitation of this study is that β-cell cAMP was measured in the bulk cytosol, which is certainly sensitive to GPCR-dependent changes, but may dilute the plasma membrane and endosomal associated cAMP signaling associated with insulin secretion with cAMP production by sAC, primarily controlled by bicarbonate generated by the mitochondrial TCA cycle. Thus, the compartmentalized cAMP signaling produced at these different subcellular locations may serve different functions. Finally, in this work we did not study the interplay between EP3 signaling and that mediated by hormones such as glucagon and GLP−1, which are now understood to have important implications for proper GSIS.^26^ª Future studies will also be necessary to understand the effects of ligand-dependent and ligand-independent EP3 receptor activity in β-cell compensation and type 2 diabetic β-cell failure.

## Materials and methods

### Animal care

C57Bl/6J *Lep*^*WT/OB*^ mice were purchased from The Jackson Laboratory and experimental mice bred in-house at the UW-Madison Research Animal Resource Center Breeding Core. WT and Gɑz-null C57Bl/6N mice have been previously described^12^ and were obtained from our in-house breeding colonies. All protocols were approved by the Institutional Animal Care and Use Committees of the University of Wisconsin-Madison and the William S. Middleton Memorial Veterans Hospital, which are both accredited by the Association for Assessment and Accreditation of Laboratory Animal Care. All animals were treated in accordance with the standards set forth by the National Institutes of Health Office of Animal Care and Use. Mice were housed in temperature- and humidity-controlled environments with a 12:12-h light/dark cycle and fed pelleted mouse chow (Laboratory Animal Diet 2920; Envigo, Indianapolis, IN) and acidified water (Innovive, San Diego, CA) ad libitum. Only male mice were used in these experiments. Blood glucose was measured by tail prick using an AlphaTRAK glucometer (Zoetis) and rat/mouse test strips. A random-fed blood glucose cutoff of 300 mg/dl was used to define hyperglycemia. Islets were isolated from experimental mice at 10–12 mice utilizing a collagenase digestion procedure as described^56^.

### Ex vivo *islet GSIS assays*

GSIS assays were performed 1 day after islet isolation as previously described.^57^ Briefly, 100 islets were washed and pre-incubated for 45 min in Krebs-Ringer Buffer containing 0.5% BSA and 3.3 mM glucose. Islets were then incubated at a density of 10 islets/ml for an additional 45 min in the indicated treatment. Secretion medium was collected, and islets were lysed in 1 ml of lysis buffer (20 mM Tris-HCl, pH 7.5; 150 mM NaCl, 1 mM Na_2_EDTA, 1 mM EGTA, 1% Triton X, 2.5 mM sodium pyrophosphate, 1 mM β-glycerophosphate, 1 mM sodium orthovanadate, and 1 μg/ml leupeptin) to determine insulin content. Insulin was assayed by ELISA as previously described.^3^

### PGE_2_ production assay

On the day of isolation, islets were cultured at a density of 50 islets/ml in normal culture media for 24 h. Secretion media was collected and cleared by centrifugation for 10 min at 10,000 g. Cleared secretion media was stored at −80°C until assayed. PGE_2_ concentrations were determined by monoclonal ELISA (Cayman Chemical #515010) following the manufacturer’s protocol as previously described.^3^

### RNA extraction, cDNA synthesis, and transcript expression analysis

RNA extraction using TRIzol (ThermoFisher), cDNA synthesis, and real-time quantitative reverse transcriptase PCR analysis (qRT-PCR) was performed using SYBR Green® reagent (Sigma Aldrich) as previously described.^58^ All gene expression was normalized to that of β-actin. Primer sequences are available upon request.

### Adenovirus amplification, purification, and transduction

A plasmid vector encoding the EPAC-based high-affinity cAMP biosensor (Epac-S^H187^) was a kind gift of Dr. Kees Jalink from the Netherlands Cancer Institute.^25^ This sensor was cloned into an adenoviral vector under the control of the rat insulin promoter in order to generate a β-cell-specific expression construct, as previously described and validated.^59^ Briefly, H187 was inserted downstream of the rat insulin 1 promoter and rabbit β-globin intron (RIP-BGI). Clonase II was used to prepare the full-length adenoviral construct in pAd-PL/DEST (Invitrogen), yielding pAd-RIP1-Epac-S^H187^-pA. Adenoviruses were amplified in HEK293 cells and purified by CsCl gradient ultracentrifugation as previously described^26^ Immediately after isolation, islets were treated with the high-titer virus (0.5 µl/ml) for 2–3 h and then returned to the islet growth medium as previously described.^26^ The adenovirus bicistronically expressing HA-tagged EP3_γ_ and GFP has been previously described.^31^ All assays utilizing adenovirus were performed 3 days after transduction.

### 2-Photon imaging of Epac-S^H187^ expression

Islets expressing pAd-RIP1-Epac-S^H187^-pA were imaged in No. 1.5 glass-bottom dishes on a multiphoton laser scanning system based around a Nikon TE−300 inverted microscope equipped with a 40×/1.15N.A. water immersion objective in a standard imaging solution (135 mM NaCl, 4.8 mM KCl, 5 mM CaCl_2_, 1.2 mM MgCl_2_, 10 mM HEPES, 10 mM glucose; pH 7.35). Temperature was maintained at 37°C with a Tokai Hit incubator. The Venus fluorescent protein was excited with a Chameleon Ultra laser (Coherent) at 920 nm. Fluorescence emission was captured by a Hamamatsu photomultiplier tube after passing through a 535/70 emission filter (Chroma). The images were collected at 512 × 512 resolution with a 2-µm z-step size at an optical zoom of 1.25 and dwell time of 2 µs.

### Simultaneous live cell Islet Ca^2+^ and cAMP imaging

Islets were imaged simultaneously for islet Ca^2+^ and β-cell cAMP as previously described^26^ In each experiment, two groups were imaged, with one group pre-labeled with 0.5 μg/ml DiR (Molecular Probes D12731) for 10 min. For measurements of cytosolic Ca^2+^, islets were pre-incubated in 2.5 μM FuraRed (Molecular Probes F3020) in islet media containing 9 mM glucose for 45 min at 37°C. Islets were imaged in a glass-bottomed imaging chamber (Warner Instruments) mounted on a Nikon Ti inverted microscope equipped with a 20×/0.75NA SuperFluor objective (Nikon Instruments). The chamber was perfused with a standard imaging solution (described above). The flow rate was set to 0.25 ml/min by a feedback controller and inline flow meter (Fluigent MCFS EZ). The temperature was maintained at 33°C using solution and chamber heaters (Warner Instruments). Excitation was provided by a SOLA SEII 365 (Lumencor) set to 5–8% output. Single DiR images utilized a Chroma Cy7 cube (710/75×, T760lpxr, 810/90 m). Excitation (x) or emission (m) filters (ET type; Chroma Technology) were used in combination with an FF444/521/608- Di01 dichroic beamsplitter (Semrock) as follows: FuraRed, 430/20× and 500/20×, 630/70 m (R430/500), for cAMP biosensor FRET imaging, CFP excitation was provided by an ET430/24× filter and emission filters for CFP and Venus emission (ET470/24 m and ET535/30 m, Chroma) reported as an emission ratio (R470/535). Fluorescence emission was collected with a Hamamatsu ORCA-Flash4.0 V2 Digital CMOS camera every 6 s. A single region of interest was used to quantify the average response of each islet using Nikon Elements. Duty cycle analysis was done utilizing custom MATLAB software (Mathworks) as previously described.^60^

### Western blot

Western blot of islet lysates was performed as previously described.^31^ Briefly, approximately 150 islets were lysed per sample, protein concentrations determined by BCA assay, and up to 30 μg protein loaded per lane. Immunoblotting was performed with rat anti-HA (Santa Cruz Biotechnology sc−53516; 1:1000 dilution), mouse anti-β-actin (Cell Signaling Technology #4970; 1:1000 dilution), rabbit anti-Rap1GAP (EMD/Merck ABIN3186690; 1:1000 dilution), or Phospho-PKA substrate (Cell Signaling Technology #9624; 1:1000 dilution) according to standard protocols. The membrane was stripped in-between the Western blot for HA and β-actin using an acidic glycine stripping buffer. Proteins were detected by enhanced chemiluminescence.

### cAMP production assay

cAMP production assays were performed as previously described (44). Briefly, on the day of assay, islets were hand-picked from RPMI into a dish containing 3 mM glucose KRBB and 15 to 20 islets per replicate picked into microfuge tubes containing 1 ml KRBB. The tubes were pre-incubated for 45 min in a 37°C tissue culture incubator, followed by a second 45 min incubation in KRBB containing 11 mM glucose and 200 μM 3-Isobutyl−1-methylxanthine (IBMX). Islets were pelleted and washed in PBS. Cellular cAMP content was analyzed using a cAMP ELISA (Cayman Chemical) according to the manufacturer’s instructions. To control for day-to-day assay variation, results were normalized within each experiment to the GFP glucose control.

### Statistical analyses

Data were analyzed using GraphPad Prism v. 8 (GraphPad Software Inc., San Diego, CA). A (1) T-test or (2) one-way ANOVA, two-way ANOVA analysis followed by Tukey post-hoc correction for multiple comparisons was used as appropriate. *P* < 0.05 was considered statistically significant.
